# Editorial: “Molecular mechanisms and physiological significance of organelle interactions and cooperation—Volume II”

**DOI:** 10.3389/fcell.2022.1076670

**Published:** 2022-11-08

**Authors:** Joseph Costello, Markus Islinger, Michael Schrader

**Affiliations:** ^1^ Faculty of Health and Life Sciences, Biosciences, University of Exeter, Exeter, United Kingdom; ^2^ Institute of Neuroanatomy, Medical Faculty Mannheim, Mannheim Centre for Translational Neuroscience, University of Heidelberg, Mannheim, Germany

**Keywords:** membrane contact sites, organelles, peroxisomes, mitochondria, lipid droplets, organelle interaction

The formation of subcellular compartments is a characteristic feature of eukaryotic life. Membrane-bound organelles such as mitochondria, peroxisomes, or lipid droplets do however not function as isolated entities; they need to cooperate and to communicate with other subcellular compartments in order to support metabolic pathways and functioning of the cell as a unit. In this regard, organelle cooperation/communication events have been associated with apoptosis, antiviral defence, organelle division and biogenesis, ROS metabolism and signalling, and various metabolic pathways such as breakdown of fatty acids. Coordinated interplay between organelles is often mediated by membrane contact sites (MCS), which bring organelles in close apposition (Simmen et al., 2017; Prinz et al., 2020). MCS can contribute to the transfer of lipids, ions and metabolites for cooperative metabolic pathways, facilitate efficient signalling and communication, or contribute to the positioning of organelles within cells. Interacting proteins (or lipids) which act as tethers to bridge the opposing organelle membranes form MCS. The research field of membrane contacts and organelle interaction continues to grow rapidly. New contact sites are still being discovered and the number of tethers and proteins associated with MCS is constantly growing (Eisenberg-Bord et al., 2016; Silva et al., 2020). However, it is still not fully understood, how MCS are regulated (Kors et al, 2022a), what their physiological functions are, and how they impact on human health and disease.

The ever expanding roles of the VAPs, a group of membrane proteins which play a critical role in connecting the ER to other organelles, are reviewed by Kors et al. The authors discuss how the FFAT motif of VAPs allows interaction with over 50 confirmed binding partners and how regulation of these interactions can allow specific control of membrane contact site formation.

Liao et al. summarize the current knowledge on the function and molecular composition of lipid droplet (LD) contacts with mitochondria, lysosomes and the ER. They highlight novel unexpected functions and discuss how LD contact site formation is influenced or regulated by organelle interaction with the cytoskeleton.

Regulation of membrane contact sites by various different mechanisms is an emerging but still relatively understudied area of research. Ravi et al. explore a novel area of membrane contact site regulation, that of the role of lipid modulators in regulating organelle crosstalk by modifying the lipid composition of membranes. Specifically focusing on Phosphotidylinositol-5-phosphate-4-kinases (PI5P4Ks), which regulate phosphatidylinositol 4,5-bisphosphate (PI-4,5-P_2_) levels, they explain how regulation of lipid phosphorylation on organelle membranes is able to regulate interaction with lipid-interacting proteins.

The number of cellular processes, which involve membrane contact sites, is increasingly expanding. In line with, this Islam et al. present new hypotheses for how membrane contact sites could influence the regulation of low density lipoprotein receptor (LDLR) internalisation and degradation. They highlight several contact site proteins that appear to be involved in this process and suggest that more emphasis should be given to understanding how membrane contact sites influence specific events in endocytosis and trafficking.

Sargsyan et al. present an interesting report on the role of calcium in peroxisomes, a topic which has not been extensively explored previously. They discuss the evidence for the presence of calcium in peroxisomes and potential peroxisomal calcium-handling proteins and also speculate on how membrane contact sites with other organelles may facilitated calcium exchange.

Many peroxisomal functions require factors also relevant to other cellular compartments. Bittner et al. review proteins, which are shared by peroxisomes and other organelles. They discuss the mechanisms to achieve dual targeting, their regulation and functional consequences with a major focus on yeast and fungi. They propose that the regulation of dual targeting will further emerge as a major factor to control organelle interplay and communication.

How lipids are transferred at membrane contact sites is still not fully understood. Using a mutagenesis strategy, Wei et al. identified Vps13 as a protein, which is essential for peroxisome formation in yeast mutants with reduced peroxisome-ER contacts. The findings suggest that in the yeast *Hansenula polymorpha* Vps13 plays a redundant function in lipid transfer from the ER to peroxisomes.

Wu and van der Klei present a first detailed structure-function analysis of *Hansenula polymorpha* ER protein Pex32, a protein of the Pex23 protein family, which only occur in yeast and filamentous fungi. *Hp*Pex32 is required for the formation of peroxisome-ER contact sites. A domain analysis now revealed that the N-terminal transmembrane domain of Pex32 contains ER sorting information, whereas the C-terminal DysF domain is required to concentrate Pex32 at peroxisome-ER contacts and can associate with peroxisomes.

Li et al. discuss the impact of peroxisome-derived H_2_O_2_ on the regulation of autophagy/pexophagy. They describe our current knowledge on how H_2_O_2_ might interfere as a signalling molecule in autophagy regulation by oxidizing thiol groups in autophagy-relevant proteins. The authors propose the concept of a peroxisome-autophagy signalling axis, which might adapt the cellular organelle status to distinct metabolic or stress situations.

Van Roermund et al. investigated the metabolic communication between the peroxisomal matrix and the surrounding cytosol. They show that a heterodimer of the yeast peroxisomal ABC transporters Pxa1p and Pxa2p, which transport long chain acyl-CoAs, can as well import cytosolic ATP into peroxisomes. In contrast to the known peroxisomal ATP transporter Ant1p, the ABC transporter heterodimer does not work as an ATP—ADP/AMP antiporter system thereby increasing the flexibility of peroxisomes to increase intraorganellar ATP concentrations. The ATP transporting function is also preserved in the human peroxisomal ABC transporters ABCD1, ABCD2 and ABCD3. Moreover, yeast peroxisomes appear to harbour a third ATP importing system, as the authors further detected that the mitochondrial membrane ATP transporter Aac2p is dually targeted to mitochondria and peroxisomes in *Saccharomyces cerevisiae*. Thus, yeast peroxisomes appear to utilize three complementing ATP importing systems to adapt intraperoxisomal ATP levels to distinct metabolic states of the cell.

The reviews and research articles presented in volume II of this special topic once again demonstrate the impressive breadth of research currently being undertaken to understand the molecular mechanisms and physiological significance of organelle interactions and cooperation.
